# A levonorgestrel-releasing intrauterine system embedded in the omentum in a woman with abdominal pain: a case report

**DOI:** 10.1186/1752-1947-3-9301

**Published:** 2009-11-27

**Authors:** Asimakis Pappas, Siddesh Shambhu, Kevin Phillips, Kate Guthrie

**Affiliations:** 1 Department of Obstetrics and Gynaecology, Attikon University Hospital, 1 Rimini Street, Xaidari, Athens, 12461, Greece; 2 Department of Obstetrics and Gynaecology, Hull Royal Women and Children's Hospital, Anlaby Road, HU3 2JZ, Hull, UK

## Abstract

**Introduction:**

The Mirena intrauterine system has been licensed as a contraceptive in the United Kingdom since May 1995. The use of an intrauterine system as a primary method of contraception among women has been slowly increasing over the last few years and they now account for about 3% of contraceptive use in England. The Mirena intrauterine system now also has a license for the management of idiopathic menorrhagia. Women may be informed that the rate of uterine perforation associated with intrauterine contraceptive use is low (0-2.3 per 1000 insertions). The rate of perforation reported with the Mirena intrauterine system in a large observational cohort study was 0.9 per 1000 insertions.

**Case presentation:**

In this case report, the diagnosis of an intraperitoneal Mirena intrauterine system was noted nearly four years after its insertion, despite the patient having had a vaginal hysterectomy and admissions to hospital in the interim with complaints of abdominal pain.

**Conclusion:**

This case report demonstrates clearly that whenever there is a question of a intrauterine system having fallen out following an ultrasound scan report showing an empty uterus, clinicians should also perform an abdominal X-ray.

## Introduction

The Mirena intrauterine system (IUS) has been licensed as a contraceptive in the United Kingdom since May 1995. Recent data from the Information Centre for Health and Social Care, NHS Contraceptive Services in England suggest that the use of an IUS as a primary method of contraception among women has been slowly increasing over the last few years and now accounts for about 3% of contraceptive use [1].

The Mirena IUS now also has a licence for the management of idiopathic menorrhagia [2] and may therefore be used by women who do not require contraception. Uterine perforation is a serious, albeit rare, complication in using an intrauterine device. Women may be informed, however, that the rate of uterine perforation associated with intrauterine contraceptive use is low (0-2.3 per 1000 insertions) [3-7]. The rate of perforation reported with the Mirena IUS in a large observational cohort study was 0.9 per 1000 insertions [8].

In this case report, the diagnosis of an intraperitoneal Mirena IUS was noted nearly four years after its insertion, despite the patient having had a vaginal hysterectomy and admissions to hospital in the interim. This case report demonstrates clearly that whenever there is a question of the IUS having fallen out following an ultrasound scan report showing an empty uterus, clinicians should perform an abdominal X-ray [3].

## Case presentation

A 33-year-old British Caucasian woman, para 2, with a long standing history of menorrhagia, dysmenorrhoea and tiredness was referred by her general practitioner (GP) to the hospital in 2002. Her GP had treated for anaemia and tiredness. Her periods had been regular although in the previous few months she had bled continuously. Her periods had become heavy after tubal sterilisation in 1996. Cervical cytology had always been normal. In the past she was diagnosed with duodenal ulcer in 1996, underwent a laparoscopy for pelvic pain for suspected endometriosis in 1997 and had an appendicectomy in 1997. On investigation she was anaemic with low iron and ferritin levels. Pelvic ultrasound scan was normal. For management of her menorrhagia, she opted for Microwave Endometrial Ablation which was performed in August 2002. Upon review after four months, she complained of erratic bleeding but no pain. An ultrasound scan showed irregular endometrium. The patient was booked for hysteroscopy under general anaesthesia. The procedure was attempted in September 2003 and was abandoned due to difficulties passing the hysteroscope through the endocervical canal. The hysteroscopy was repeated in January 2004 and a few intrauterine adhesions were reported. An endometrial biopsy was done just before the insertion of a Mirena IUS under the same general anaesthetic. The results of the biopsy were normal. It was not recorded if the sitting of the IUS was checked post insertion hysteroscopically. Her GP was asked to check the threads in four to six weeks time.

A month later, she was admitted to the hospital with right upper quadrant pain and a problematic bleeding pattern. Ultrasound at this stage showed a normal size uterus but the Mirena IUS was not in situ. It was assumed the Mirena IUS had fallen out. The patient elected to have a vaginal hysterectomy to solve her bleeding problems, which was performed in September 2005.

In January 2007, she was admitted to the hospital with right upper quadrant pain again and all investigations including chest X-ray, abdominal ultrasound scan and blood tests were normal. She had an upper GI endoscopy, which showed a gastric ulcer.

**Figure 1:**
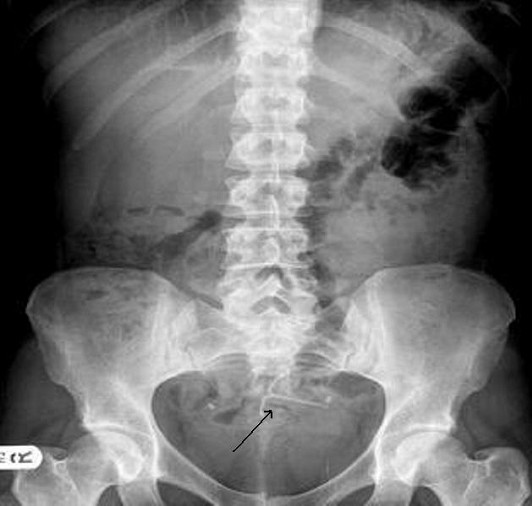
**Abdominal X-ray with the Mirena IUS (arrowed)**.

**Figure 2:**
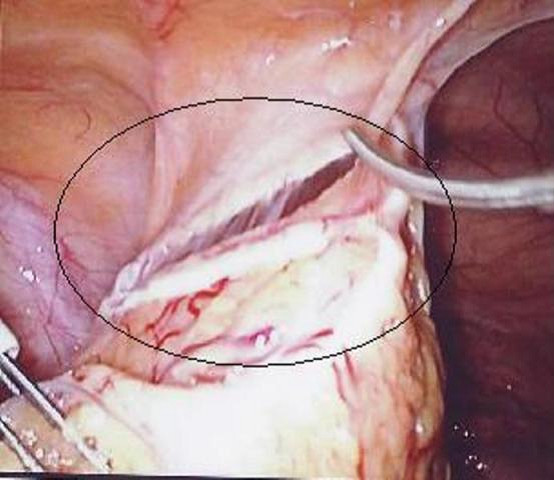
**The Mirena intrauterine system (circled) within the omentum**.

She was admitted again in April 2008 with pelvic pain. An abdominal X-ray showed the lost IUS (Figure 1) and a CT scan showed the IUS lying anteriorly under the rectus muscles and adjacent to the dome of the bladder. In April 2008 the IUS was retrieved laparoscopically. The omentum was adherent to the anterior abdominal wall and the Mirena IUS was found in the omentum, (Figure 2). The IUS was removed easily from the abdominal cavity laparoscopically. The right tube and ovary were adherent to the right pelvic wall and they were freed up. The procedure was uneventful and the patient was discharged the same day. The most likely cause of the abdominal pain in this patient was the adhesions. The patient had no complaints of abdominal pain in the 6 months of follow-up.

## Discussion

Uterine perforation is a serious, albeit rare, complication of intrauterine contraceptive use. The rate of perforation reported with the Mirena IUS in a large observational cohort study was 0.9 per 1000 insertions [8]. However, the UK Medical Eligibility Criteria for Contraceptive use [9] considers a uterine cavity distorted for any reason, congenital or acquired, to be a category 4 condition, i.e. a condition which represents an unacceptable health risk if the contraceptive method is used. The British National Formulary (BNF) [10] also suggests that intrauterine devices (IUDs) should be used with caution in severely scarred uteri. In this case, the Mirena was inserted after intrauterine surgery. The Faculty of Sexual and Reproductive Healthcare, Clinical Guidance 2007, 'Intrauterine Contraception' [3] recommends that a routine follow-up visit should be done after the first menses following insertion of intrauterine contraception or three to six weeks after the insertion. The Royal College of Obstetricians and Gynaecologists recommends [11] that women who present with persistent menorrhagia, despite Mirena IUS use, should be advised to return for further assessment of the uterine cavity, using hysteroscopy or ultrasound scan, to exclude pathology. An endometrial biopsy should be considered in all women with persistent menorrhagia who are 35 or older, because progesterone therapy may alter endometrial histology and may mask a premalignant or malignant lesion.

Endometrial ablation or resection are surgical methods to manage menorrhagia. Intrauterine scarring may occur following treatment. A narrative review paper on treatment after hysteroscopic surgery suggests that an acceptable post-operative method of contraception after endometrial ablation is the Mirena IUS, as it protects the endometrium and there is a high rate of amenorrhoea [12]. However, following successful endometrial ablation the uterine cavity is usually severely narrowed making insertion of an IUS impossible and it would not normally be considered in these circumstances. Significant bleeding would suggest failure of the procedure, and if IUS was to be considered it should only be done with hysteroscopic assistance by an experienced gynaecologist [13]. A thorough literature search of the Medline, Embase and Cochrane databases did not reveal any similar case reports and also did not report any formal guidance to the use of the Mirena IUS device following endometrial microwave ablation. There was one article, though, regarding insertion of Mirena IUS, after endometrial resection [14].

Generally, the presence of risk factors should always be identified before the insertion of IUDs. Risk factors include the postpartum period or during breast feeding, a retroverted uterus, uterine anomalies and inexperience of the operators which can increase the risk of perforation [15].

In summary, in this case report, the diagnosis of the Mirena IUS inside the peritoneal cavity was noted nearly four years after the insertion and was presumed to be due to perforation or partial perforation of the uterus. The patient had several admissions to hospital under the care of gynaecologists or gastroenterologists complaining for upper or lower abdominal pain. She even had a vaginal hysterectomy. Had she undergone an abdominal hysterectomy, the Mirena IUS may have been noted at that time.

This case report demonstrates the need for regular IUS thread checks and that following an ultrasound report showing an empty uterus in a symptomatic patient, an abdominal X-ray should be performed to identify whether or not the IUS is inside the peritoneal cavity. We advocate that following basic rules after IUS insertion, such as hysteroscopic insertion post ablation, annual check-up of IUS position, X-ray for a patient complaining of abdominal pain will prevent complications or assist in their early diagnosis.

## Abbreviations

IUS: intrauterine system; IUD: intrauterine device.

## Competing interests

The authors declare that they have no competing interests.

## Authors' contributions

AP: Main surgeon, main writer of the case report; SS: co-writer; KP: reviewer of the case report; KG: co-writer, reviewer of the case report, literature research.

## Consent

Written informed consent could not be obtained because the patient was lost to follow-up. Despite repeated attempts we were unable to trace the patient or her family. We believe this case report holds a worthwhile clinical lesson which could not be communicated effectively in any other way. Every effort has been made to keep the patient's identity anonymous. We would not expect the patient or their family to object to publication.
